# Clinical Utility of Virtual Kitchen Errand Task for Children (VKET-C) as a Functional Cognition Evaluation for Children with Developmental Disabilities

**DOI:** 10.3390/children11111291

**Published:** 2024-10-25

**Authors:** Yumi Ju, Sura Kang, Jihye Kim, Jeh-Kwang Ryu, Eun-Hwa Jeong

**Affiliations:** 1 Major in Human Development and Rehabilitation, Graduate School of Education Service Science, Dongguk University, Seoul 04620, Republic of Korea; zxc83@dongguk.edu (Y.J.); ryujk@dgu.ac.kr (J.-K.R.); 2 Center of Convergence Research in Artificial Intelligence, Dongguk University, Seoul 04620, Republic of Korea; surakang7410@dongguk.edu; 3 Mustard Seeds Center of Child Development, Cheong-Ju 28425, Republic of Korea; wisdom12391@gmail.com; 4 Department of Physical Education, Dongguk University, Seoul 04620, Republic of Korea; 5 Department of Occupational Therapy, Kyung-in Women’s University, Incheon 21041, Republic of Korea

**Keywords:** children, functional cognition, performance error, virtual kitchen errand task, virtual reality

## Abstract

Background/Objectives: This study evaluated the clinical utility of a virtual reality (VR)-based kitchen error task for children (VKET-C) to assess functional cognition in children. Methods: In total, 38 children aged 7–12 years were included, comprising 23 typically developing (TD) children and 15 children with developmental disabilities (DDs), including autism spectrum disorder, attention deficit hyperactivity disorder, and intellectual disability. While performing the VKET-C, performance errors were analyzed. The Stockings of Cambridge (SOC) and Spatial Working Memory (SWM) tasks from the Cambridge Neuropsychological Test Automated Battery (CANTAB) were used to assess cognitive function. The Brunner–Munzel test was performed to compare performance errors between the TD and DD groups, and correlations between performance errors and cognitive measures were analyzed. Results: Omission and commission errors were significantly different between the groups (*p* < 0.001), with no significant difference in motor errors (*p* > 0.05). Omission errors were correlated with the initial thinking time mean (ITMN) in all items of the SOC task and the between errors (BE) of the SWM task. Commission errors were correlated with the ITMN in the difficult items of the SOC task and the BE of the SWM task. Additionally, motor errors were significantly correlated with problems solved in minimum moves (PSMM) and ITMN in the difficult items of the SOC task and BE in the SWM task. Conclusions: The VKET-C shows promise as an effective tool for assessing executive function and working memory in children with DDs, offering an engaging and ecologically valid alternative to traditional methods.

## 1. Introduction

Virtual reality (VR) has become a useful medium for delivering assessments and interventions in rehabilitation [1,2]. VR-based assessments have the advantage of creating a context-specific environment for assessing functional cognition. They are considered ecological assessments with a high degree of veridicality and verisimilitude compared to traditional pencil and paper assessments [3,4]. The performance outcomes of a VR task predict how one’s cognitive functions operate in daily life and can be used to identify deficits. In the recent literature, VR has commonly been used to assess visuospatial navigation, episodic memory, and executive function to detect neurological deficits in older populations [5,6]. These cognitive domains manifest in complex contexts where individuals interact with their environments and objects. VR offers the advantage of creating virtual environments that are both relevant and immersive without the constraints of physical space or cost [7].

VR technology has attracted interest in its potential as a therapeutic tool, including serious games, as it is widely used in education and entertainment for children [8,9,10]. Children’s acceptance of VR technologies is higher than that of adults, and a VR environment can provide a safe and structured environment for children [11]. VR has been used to evaluate daily living skills and social interaction skills for children with autism spectrum disorders (ASDs) and intellectual disability (ID) [12,13]. An avatar can serve as a social partner or role model, providing children with disabilities with the advantage of repetitive practice opportunities. In addition, virtual environments help prevent real-world issues and avoid the humiliating or dangerous consequences of errors [13].

Functional cognition is important for predicting a child’s primary participation and health in daily life [14]. It is conceptualized as a cognitive ability that incorporates metacognition, executive function, problem-solving, and other cognitive domains [14,15]. Among them, executive functioning plays a central role in harmonizing these elements in daily task performance; it is the skill of establishing the goal for behaviors, planning strategies to achieve the goal, and self-monitoring during activities [16]. Previous research has demonstrated that executive function serves as the foundation for instrumental activities of daily living (IADL), such as vocational tasks in the workplace and even financial management, as children grow [17]. Thus, accurately assessing and monitoring executive function across the lifespan is essential to promoting engagement in daily life with independence.

Executive impairments are commonly observed in children with attention deficit hyperactivity disorder (ADHD), ASD, ID, and other neurological conditions, and are a critical factor that interferes with their performance in daily life and school [18]. Executive function is generally assessed by observing the performance of complex and demanding tasks that require a specific sequence [19]. Occupation-based assessments, such as the Children Cooking Task (CCT), Do-Eat Assessment, Preschool Executive Task Assessment (PETA), and Children’s Kitchen Task Performance (CKTA), are standardized assessments of executive function in the natural context for children [20,21].

The kitchen task is a familiar daily task. It includes a series of complex processes, such as goal setting, planning, organization, sequencing, error monitoring, and correction [22]. A study utilizing kitchen tasks demonstrated that performance was highly correlated with measures of memory, working memory, and executive function [23,24]. Observing the performance errors while performing the kitchen task was considered a remarkable index of cognitive decline or problems. Performance errors can commonly be classified into substitution, spatial misorientation/misestimation, omission, perseveration, and action addition [25]. Giovannetti et al. (2008) developed a framework for performance errors as an assessment index. They classified performance errors as omission errors (type 2 errors, or misses) or commission errors (type 1 errors, or false alarms) [26]. Omission errors refer to errors in omitting steps when performing a task, whereas commission errors include substitution, pace, and spatial errors, which include errors in adding or replacing steps.

Previous studies have attempted to assess cognitive impairment by measuring omission and commission errors during the performance of kitchen tasks [5,25]. Omission errors are related to episodic memory and executive control [27,28]. The Continuous Performance Test (CPT), a neuropsychological assessment tool used as a diagnostic indicator for children with ADHD, has also been adopted as a framework for assessing behavioral errors [29,30,31]. Omission errors are indicative of inattention, whereas commission errors reflect impulsivity [31]. Accordingly, analyzing performance errors in daily tasks could provide a more comprehensive interpretation of deficits in functional cognition than merely measuring success, failure, and execution time. However, to the best of our knowledge, few studies have evaluated cognitive function in the pediatric population by observing performance errors during daily tasks.

Given the growing interest in utilizing VR for the ecological assessment of functional cognition within naturalistic contexts, evaluating the clinical utility of VR tasks in the pediatric population is crucial. Specifically, it is necessary to determine whether VR-based assessments can effectively differentiate between children with a broad range of developmental disabilities (DDs) and typically developing (TD) children. This study aimed to apply a VR-based kitchen errand task to TD children and children with DDs and analyze the performance errors observed during the kitchen errand task. Additionally, this study aimed to verify whether performance errors correlate with cognitive function, as measured by a tablet-based computerized neuropsychological test.

## 2. Materials and Methods

### 2.1. Participants

In total, 41 school-age children (aged 7–12 years) initially participated in this study. Of these, 23 were TD children, and 18 were children with DDs diagnosed with ASD (*n* = 4), ADHD (*n* = 2), and ID (*n* = 9). A physician diagnosed participants in the DD group. They were enrolled in the Ministry of Health and Welfare to receive vouchers for rehabilitation from the developmental rehabilitation services and community social service investment project. TD children were randomly recruited from open sources, ensuring diversity and reducing selection bias. Children with DDs were recruited from four different clinics in various regions. In the DD group, three children (16.6%) were excluded because of difficulties in in-depth perception and controller manipulation; thus, only 15 children were included in the DD group. No dropouts were reported in the TD group. Participants were recruited through open social network services and community-based after-school sessions. Written informed consent was obtained from both the children and their parents, and the participants were provided with appropriate compensation. This study was approved by the Institutional Review Board of Dongguk University (DUIRB-20231026).

### 2.2. Apparatus and Measurements

#### 2.2.1. Virtual Kitchen Errand Task in Children (VKET-C)

**Figure 1:**
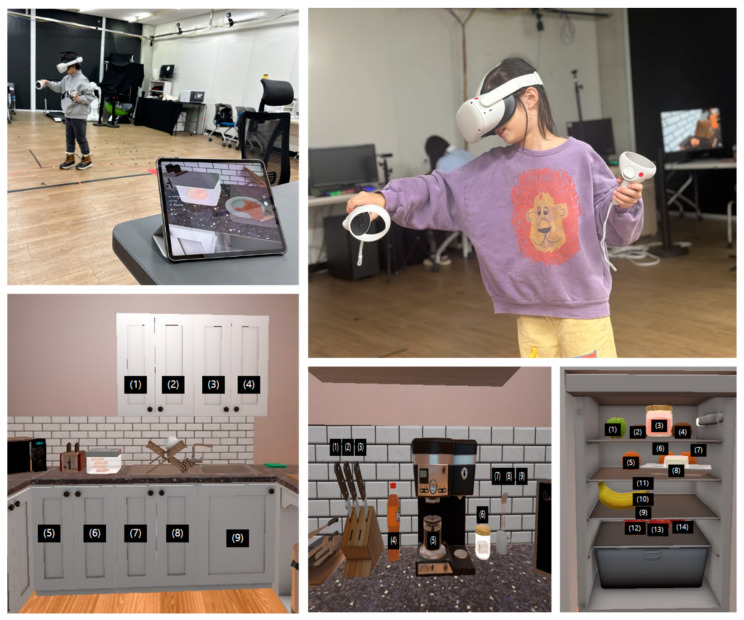
Scene for children performing the VR task and virtual items in the virtual kitchen.

The VKET-C was designed to assess functional cognition in children using a head-mounted display (HMD)-based VR (Oculus Quest2) (Figure 1). There were three task requirements: (1) Putting two pieces of bread on a plate, (2) pouring the brewed coffee into a mug, (3) taking a banana from the refrigerator, and placing all three on a table. This task included working memory to remember items and executive functions to plan and organize efficient sequences to be performed in the shortest possible time. The participants noted the location of the objects, and pretraining for controller manipulation was provided until they had proficiently manipulated the controllers. The participants were instructed to perform the task accurately and as quickly as possible.

#### 2.2.2. Scoring Performance Errors

Performance errors were measured while the tasks were performed. There were three types of errors: omission errors (task completion by step omission), commission errors (substitution, perseveration, sequence errors, addition, and micro-errors), and motor errors (mis-operation of controllers) [24,32] (Table 1). Two occupational therapists who repeatedly observed video recordings of the participants’ performances evaluated performance errors until an agreement was reached.

### 2.3. Cambridge Neuropsychological Test Automated Battery (CANTAB)

The CANTAB is a tablet-based standardized neurocognitive assessment developed by Cambridge Cognition [33]. It has subsets that can evaluate various cognitive domains, in which the Stockings of Cambridge (SOC) is used for executive function, and the Spatial Working Memory (SWM) tasks were used for spatial working memory.

The SOC task involved making the same arrangement of balls as shown above with minimum movement. The measured variable was the average initial thinking time to plan movements before starting the task (initial thinking time mean, ITMN), and the level was divided from two to five, which indicated the number of moves. The second variable was the number of assessed problems that the participant successfully completed within the minimum number of moves (problems solved in minimum moves, PSMM). It took approximately 10 min to complete the evaluation.

The SWM task involved finding a token in a box by remembering its location. The measured variable included the number of incorrectly revisited boxes in which the token had already been found (between errors; BE). It took approximately 5 min to complete the evaluation. The exact operational definitions of these variables are listed in Table 2.

### 2.4. Statistical Analysis

All statistical analyses were performed using Jamovi 2.3.18. Descriptive statistics were used to analyze participant characteristics. The variance in performance errors between the TD and DD groups was not equal, and the data did not follow a normal distribution; therefore, the Brunner–Munzel test was used to compare the two groups [34]. Pearson’s correlation analyses between the performance errors and SWM and SOC scores from the CANTAB were also conducted.

## 3. Results

### 3.1. Demographic Characteristics

The final data from 38 children (TD, *n* = 23; DD, *n* = 15) were analyzed. The average age of the TD group was 8.91 years (SD = 1.44), while that of the DD group was 9.80 years (SD = 1.93). There was no statistically significant difference in age between the TD and DD groups (*p* = 0.114). In the TD group, 11 boys (47.83%) and 12 girls (52.17%) had equivalent sex ratios (*p* = 0.402). In the DD group, there were five boys (33.33%) and ten girls (66.67%), with a higher proportion of girls, although this was not statistically significant (*p* = 0.378). The ethnicity of the participants in groups was Korean. In the TD group, 20 children (89.98%) lived in urban areas and 3 (13.04%) lived in rural areas. In the DD group, 15 children (100%) lived in Metropolitan cities (Table 3).

### 3.2. Comparison of Performance Errors Between the TD and DD Groups

To compare performance errors between the TD and DD groups, the Brunner–Munzel test was conducted. The results showed that mission and commission errors were significantly different between the TD and DD groups (*p* < 0.001); however, there was no statistically significant difference in motor errors (*p* > 0.05) (Table 4).

The Intraclass Correlation Coefficient (ICC) for the initial scoring was 0.831 (95% CI: 0.800–0.858). The scores were agreed upon after a discussion following the repeated observation of the recorded video.

### 3.3. Frequency of Performance Errors According to Diagnosis

The frequency of performance errors according to the type of diagnosis was analyzed.

Omission errors were the lowest in the TD group (mean: 0.17, SD: 0.83), while omission errors in the other diagnostic groups ranged from 1.50 to 1.56. Commission errors most frequently occurred in the ADHD group (mean: 7.00, SD: 4.24), followed by the ASD (mean: 6.25, SD: 3.20), ID (mean: 5.11, SD: 2.57) and TD (mean: 2.48, SD: 1.81) groups. Motor errors were the lowest in the TD group (mean: 1.26, SD: 1.01), while motor errors in other diagnostic groups ranged from 2.00 to 2.56. The results of the CANTAB sub-items—ITMN, PSMMT, and BE—according to diagnostic groups are shown in Table 5.

### 3.4. Correlation Between Performance Errors and the CANTAB Results

The correlations between the performance errors observed during the kitchen errand task and the results of the CANTAB were analyzed. Omission errors showed a significant positive correlation with SWM BE (0.339, *p* = 0.013) and statistically significant negative correlations with SOC ITMN2 (–0.371, *p* = 0.022), SOC ITMN3 (–0.349, *p* = 0.032), SOC ITMN4 (–0.569, *p* < 0.001), SOC ITMN5 (–0.494, *p* = 0.002), and SOC PSMMT (–0.613, *p* < 0.001) (Table 6).

Additionally, commission errors showed a significant positive correlation with the SWM BE (0.472, *p* = 0.003) and statistically significant negative correlations with SOC ITMN4 (–0.435, *p* = 0.006) and SOC ITMN5 (–0.402, *p* = 0.012). Finally, motor errors showed a significant positive correlation with the SWM BE (0.327, *p* = 0.045) and a significant negative correlation with the SOC ITMN4 (–0.046, *p = 0*.004), SOC ITMN5 (–0.327, *p* = 0.045), and SOC PSMMT (–0.527, *p* < 0.001) (Table 6).

## 4. Discussion

This VR-based cognitive assessment has been considered an alternative to traditional pencil-and-paper evaluations for assessing functional cognition [35]. It has the advantage of constructing an evaluation environment without constraints and organizing systematic and consistent task contexts in every session [36,37,38]. In addition, most participants positively accepted and enjoyed performing the tasks in VR. The interactive nature of VR increases children’s motivation to participate in assessments [39,40]. In a previous study, the acceptance of VR technology for children with disabilities was found to be positive in VR-based activities [41]. Although it was somewhat anticipated that children with autism might experience discomfort due to unfamiliar sensory experiences in VR, it was found that these experiences did not trigger any issues related to sensory sensitivity [42]. In the present study, no child resisted the virtual environment due to sensory discomfort while performing the VR task.

The results of this study indicate that children with DDs exhibit omission and commission errors more frequently than TD children. Previous research has also shown that children with neuropsychological impairments tend to make more omission and commission errors when performing cognitive tasks than TD children [43,44,45]. Children with DDs commonly demonstrate difficulties in executive functioning, including deficits in working memory, planning, sequencing, inhibition, and cognitive flexibility. These deficits can lead to various performance errors during task execution [46,47]. Executive functioning refers to the ability to maintain the goals of a behavior and utilize information while performing tasks [21]. Difficulties in executive function and working memory can result in an inability to maintain the task goal, leading to omission errors and subsequently impacting task completeness [48,49]. Children with poor self-regulation, such as those with traumatic brain injury (TBI) or ADHD, are known to exhibit a tendency to skip essential steps during task performance [50,51,52].

In the VKET-C, omission errors exhibited by children were correlated with all variables (ITMN 2–5, PSMMT) of the SOC test and the BE measure of the SWM test. The ITMN during the SOC task was negatively correlated with omission errors, regardless of task difficulty. In other words, shorter initial thinking time in the SOC task was associated with a higher frequency of omission errors. Additionally, the number of successful attempts in the PSMMT was negatively correlated with omission errors in the virtual kitchen task. Those results indicate that children exhibiting more frequent omission errors in the VKET-C may demonstrate poor planning and executive control abilities. In the SOC task results, children with ASD exhibited shorter initial thinking times and fewer successful trials compared to those with TD. However, while children with ADHD also showed fewer successful trials, they did not demonstrate shorter initial thinking times; instead, they showed longer initial thinking times on certain items (ITMN 2, 3, 5). Children with ADHD and ASD both showed difficulties in executive function; however, the patterns of executive deficits slightly differed between the two groups [53]. Children with ASD showed greater deficits in planning, selective attention, and attentional shifting [53]. Our results suggest that children with ASD tend to have poorer planning abilities compared to those with ADHD.

Omission errors were also correlated with the BE measure in the SWM, indicating that a higher frequency of omission errors was associated with more errors in remembering the location of a previously presented token in the SWM task. Omission errors are closely linked to deficits in working memory [54,55]. Children’s working memory capacity is particularly susceptible to memory decay during online processing [54]. When the decay of information that needs to be retained during task performance is pronounced, children may forget essential steps, compromising the overall quality of task completion. Previous studies utilizing a combined N-back/nogo paradigm in children with ADHD reported a high frequency of omission errors and neurophysiological evidence pointing to working memory deficits [55]. While prior research predominantly employed tabletop tasks, the present study extends these findings by demonstrating that omission errors in children with DDs are also associated with working memory in a more ecologically valid setting, such as a virtual kitchen task.

A commission error encompasses substitution, perseveration, sequence errors, addition, and micro-errors. In the results, commission errors were found to have a negative correlation with the ITMN at the difficult levels (ITMN 4 and 5) of the SOC task. Specifically, the ITMN was not associated with commission errors for easier items (ITMN 2 and 3); however, a shorter ITMN for more difficult items was significantly associated with a higher frequency of commission errors. This may be attributed to the increasing importance of planning as the complexity of SOC tasks increases. Furthermore, commission errors were positively correlated with BE in the SWM task, suggesting that this error may also be related to working memory. According to Matos et al., as the cognitive load of the ongoing task increases, the intention associated with prospective memory is not disengaged, which can lead to commission errors [56]. The findings of this study similarly indicate a positive correlation between task difficulty and the occurrence of commission errors. Those findings suggest that commission errors are influenced by the task’s complexity and working memory capacity.

In the present study, the group of children with DDs showed a significantly higher occurrence of commission errors than the TD children. Notably, children with ADHD and ASD exhibited a higher frequency of commission errors compared to the ID group. Commission errors involve displaying inefficient or problematic behaviors that should not occur—in other words, false-positive behaviors [29]. Due to the immature development of cognitive functions, commission errors occur more frequently in task performance, reflecting impulsivity [52]. However, robust statistical analyses could not be performed due to the small number of children recruited for each diagnostic group, which is a limitation of this study.

In contrast to omission and commission errors, motor errors did not differ significantly between the TD and DD groups. A motor error was defined as a child repeatedly acting to handle or drop objects owing to a lack of manipulating controllers. The difference in the hand manipulation errors observed between the two groups was not statistically significant. In this study, the kitchen errand task was designed to minimize the impact of controller manipulation. During task performance, children were only required to grasp and grab the cabinet knobs or targeted objects (e.g., bread, plate, coffee pot, cup, banana); no additional motor actions were necessary. Previous studies on the usability of various controller manipulation methods have shown that grasping knob-type objects in virtual reality is most similar to real hand movements and is considered the easiest and most preferred method by participants [57]. Children with DDs often display motor clumsiness, which can result in underestimating their cognitive abilities in assessments requiring motor responses [58,59]. However, this study demonstrates that using VR to assess cognitive function minimizes the impact of motor clumsiness, leading to more accurate evaluations.

This study also highlights important considerations for VR research on children with DDs. First, the dropout rate among the children with DDs was 16.6%, which may be because some children are not well suited to VR, especially those with difficulties with depth perception or fine motor manipulation. Further research should explore the adaptations to the VR environment in which children perceive and interact with the virtual world. Alternative input devices can be used to reduce these barriers and make VR assessments more accessible to a broader range of participants. Nevertheless, VR has the advantage of providing an interactive and engaging nature to children, and a child’s cognitive functions can be assessed in the real context of activities [39]. Previous studies on VR-based assessments highlight the advantages of early identification of cognitive dysfunction [5,6]. Tragantzopoulou and Giannouli [60] emphasized that VR-based spatial orientation assessments offer a more ecologically valid approach, enhancing patient motivation and the sense of immersion in task performance. These assessments have demonstrated improved diagnostic precision in the early stages of neurological diseases [60]. The VKET used in this study is anticipated to be particularly effective for identifying borderline cognitive functioning in children. It also provides an advantage over traditional pencil and paper assessments, which often face challenges when children fail to comply with evaluation instructions.

In summary, this study highlighted the possibility of using the VKET-C to evaluate functional cognition in children with DDs. As a result, false-positive and false-negative behaviors in the VKET-C were significantly related to executive function and working memory. Further research is needed to validate these findings in larger populations to validate their clinical application. Additionally, recruiting a sufficient number of participants based on their diagnoses would allow for a more detailed understanding of the subtypes of the cognitive characteristics associated with different diagnoses. Moreover, integrating physiological measures, such as eye tracking and heart rate variability, could provide additional insights into attentional and emotional regulation during VR task performance. This approach can enhance the application of VR in addressing children’s health and developmental issues.

## 5. Conclusions

This study demonstrates the potential of the VKET-C for evaluating functional cognition in children with DDs. The findings showed that omission and commission errors in the VR tasks were significantly associated with executive function and working memory, suggesting that VR tasks can effectively identify cognitive deficits. In addition, there was a statistically significant difference in the frequency of errors between children with DDs and those with TD. While challenges, such as the dropout rate among children with DDs, were noted, future research should explore adaptations to VR environments and alternative input devices to improve the accessibility of VR. Further validation with a larger population is also needed, and we hope that the VKET-C can be used to address children’s developmental needs.

## Figures and Tables

**Table 1:** Types of performance error and its description.

Error Type	Description	Example
Omission	Missing step	Bring only one piece of bread
		Do not put bread on the plate
		Not closing the lid/door
		Missing one of target items (coffee, banana, bread)
Substitution	Different tools were used	Pour the coffee in other objects
		Put the bread in other objects
Perseveration	Repeating steps without reason	Bringing more than the specified quantity
		Moving spot to spot without a purpose
Sequence Error	Changing the order of steps in an inefficient way	Take out bread first without a plate
		Do not bring a mug and grab a coffee pot
		Take out a plate/cup/bread and do not close the door right away, and close it later after going somewhere else
Addition	Adding unnecessary steps	Put a banana on a plate
		Bring unnecessary things
Micro-Error	Touching objects without purpose	Touch objects for no reason
Motor Error	Lack of manual dexterity	Repeat an action (more than three times) due to difficulties in grasping or closing a door
		Dropping an object

**Table 2:** Definition of variables on SOC and SWM of CANTAB.

Test	Sub-Item Title	Description	Level
SOC (Stockings of Cambridge)	ITMN (Initial Thinking Time Mean)	Initial thinking time is the difference in the time taken to select the first ball for the same problem in the solve compared to follow conditions.	2, 3, 4, 5
	PSMM (Problems Solved in Minimum Moves)	The number of assessed problems that the subject successfully completed in the minimum possible number of moves. Calculated over all assessed trials	Total
SWM (Spatial Working Memory)	BE (Between Errors)	The number of times the subject incorrectly revisits a box in which a token has previously been found.	Total

**Table 3:** Characteristics of participants.

Categorization		TD (*n* = 23)	DD (*n* = 15)	*p*
Age (Mean (SD))		8.91 (1.44)	9.80 (1.93)	0.114
Gender (*n* (%))	Boys	11 (47.83)	5 (33.33)	0.402 (TD)/0.378 (DD)
	Girls	12 (52.17)	10 (66.67)	
Ethnicity (*n* (%))	Korean	23 (100)	15 (100)	
Residence (*n* (%))	Metropolitan	20 (89.96)	15 (100)	
	Rural Area	3 (13.04)	0 (0)	

**Table 4:** Comparison of TD and DD in performance errors.

		Median	IQR	Statistic	*df*	*p*	Effect Size
Omission Errors	TD	0	0.00	−4.37	21.6	<0.001	0.19
	DD	1	2.50				
Commission Errors	TD	2	3.00	−5.28	33.7	<0.001	0.16
	DD	5	4.00				
Motor Errors	TD	1	1.00	−2.03	20.2	0.056	0.30
	DD	2	2.00				

IQR: interquartile range; Note. Effect size = $\hat{P}$ (DD < TD) + 1/2$\hat{P}$ (DD = TD).

**Table 5:** Frequency of performance errors according to diagnosis type.

Diagnosis (*n* = 38)		TD (*n* = 23)	ADHD (*n* = 2)	ASD (*n* = 4)	ID (*n* = 9)
Omission Errors	Mean (SD)	0.17 (0.83)	1.50 (0.70)	1.50 (2.38)	1.56 (1.51)
Commission Errors	Mean (SD)	2.48 (1.81)	7.00 (4.24)	6.25 (3.20)	5.11 (2.57)
Motor Errors	Mean (SD)	1.26 (1.01)	2.00 (0.00)	2.50 (2.52)	2.56 (2.01)
SOC ITMN2	Mean (SD)	1542 (919)	1823 (619)	672 (260)	1163 (1584)
SOC ITMN3	Mean (SD)	3569 (2749)	7887 (7163)	1224 (582)	901 (737)
SOC ITMN4	Mean (SD)	4561 (3052)	2577 (1866)	1427 (1140)	2925 (2673)
SOC ITMN5	Mean (SD)	3235 (2819)	3513 (1647)	1079 (1121)	1425 (1032)
SOC PSMMT	Mean (SD)	6.48 (1.38)	4.50 (2.12)	4.50 (1.29)	3.11 (2.67)
SWM BE	Mean (SD)	13.70 (8.99)	27.50 (3.54)	22.50 (3.00)	25.20 (5.91)

**Table 6:** Results of correlation analysis.

		Omission Errors	Commission Errors	Motor Errors
Omission Errors	Pearson’s r	-		
	*p*-value	-		
Commission Errors	Pearson’s r	0.390 *	-	
	*p*-value	0.015	-	
Motor Errors	Pearson’s r	0.651 ***	0.365 *	-
	*p*-value	<0.001	0.024	-
SOC ITMN2	Pearson’s r	−0.371 *	−0.111	−0.058
	*p*-value	0.022	0.507	0.728
SOC ITMN3	Pearson’s r	−0.349 *	−0.290	−0.208
	*p*-value	0.032	0.078	0.210
SOC ITMN4	Pearson’s r	−0.569 ***	−0.435 **	−0.460 **
	*p*-value	<0.001	0.006	0.004
SOC ITMN5	Pearson’s r	−0.494 **	−0.402 *	−0.327 *
	*p*-value	0.002	0.012	0.045
SOC PSMMT	Pearson’s r	−0.613 ***	−0.320	−0.527 ***
	*p*-value	<0.001	0.050	<0.001
SWM BE	Pearson’s r	0.399 *	0.472 **	0.327 *
	*p*-value	0.013	0.003	0.045

* *p* < 0.05, ** *p* < 0.01, *** *p* < 0.001; ITMN: Initial Thinking Time Mean, PSMMT: Problems Solved in Minimum Moves Total, BE: Between Errors.

## Data Availability

Data are available from the DGU Institutional Data Access/Ethics Committee (contact via Yumi Ju (catharina.ymj@gmail.com) for researchers who meet the criteria for access to confidential data).
